# Sleep SAAF: a responsive parenting intervention to prevent excessive weight gain and obesity among African American infants

**DOI:** 10.1186/s12887-019-1583-7

**Published:** 2019-07-05

**Authors:** Justin A. Lavner, Brian K. Stansfield, Steven R. H. Beach, Gene H. Brody, Leann L. Birch

**Affiliations:** 1 Department of Psychology, University of Georgia, Athens, USA; 2 Department of Pediatrics, Augusta University, Augusta, USA; 3 Department of Psychology and Center for Family Research, University of Georgia, Athens, USA; 4 Center for Family Research, University of Georgia, Athens, USA; 5 Department of Foods and Nutrition, University of Georgia, Athens, USA

**Keywords:** Rapid weight gain, Obesity, Prevention, Infancy, Responsive parenting, African Americans

## Abstract

**Background:**

Responsive parenting interventions that shape parenting behaviors in the areas of sleep and soothing, appropriate and responsive feeding, and routines represent a promising approach to early obesity prevention and have demonstrated effectiveness in our previous trials. However, this approach has yet to be applied to the populations most at-risk for the development of early obesity, including African Americans. The Sleep SAAF (Strong African American Families) study is a two-arm randomized controlled clinical trial evaluating whether a responsive parenting intervention focused on promoting infant sleeping and self-soothing can prevent rapid weight gain during the first 16 weeks postpartum among first-born African American infants. The responsive parenting intervention is compared to a child safety control intervention.

**Methods:**

Three hundred first-time African American mothers and their full-term infants will be enrolled from one mother/baby nursery. Following initial screening and consent in the hospital, mothers and infants are visited at home by Community Research Associates for data collection visits at 1 week, 8 weeks, and 16 weeks postpartum and for intervention visits at 3 weeks and 8 weeks postpartum. The primary study outcome is a between-group comparison of infant conditional weight gain (CWG) scores from 3 weeks to 16 weeks; additional weight-related outcomes include differences in change in infants’ weight for age over time and differences in infants’ weight outcomes at age 16 weeks. Several other outcomes reflecting infant and maternal responses to intervention (e.g., sleeping, soothing, feeding, maternal self-efficacy, maternal depressive symptoms) are also assessed.

**Discussion:**

The Sleep SAAF trial can inform efforts to prevent rapid weight gain and reduce risk for obesity early in the lifespan among African Americans.

**Trial registration:**

NCT03505203. Registered April 3, 2018 in clinicaltrials.gov.

**Electronic supplementary material:**

The online version of this article (10.1186/s12887-019-1583-7) contains supplementary material, which is available to authorized users.

## Background

Racial disparities in obesity begin early in development and continue throughout the lifespan [1, 2]. From birth to 2, the prevalence of high weight for length (above the 95th percentile) is 25% higher among African American children compared to White children [1]. From age 2–19, the rate of obesity is more than 50% higher among African American children compared to White children [3]. Similar disparities persist into adulthood: rates of obesity are approximately 25% higher among African American adults compared to White adults [1]. Differences in early rapid weight gain during infancy (RWG) may be a key driver of these disparities: RWG is one of the most consistent factors associated with later overweight and obesity [2, 4–14], is more common among African American infants than among White infants [2, 15], and accounts for more than 70% of the difference between White and Black children’s BMI z scores during early childhood [2].

The higher prevalence of RWG and actual childhood obesity rates among African Americans suggest that intervening early in development has great potential to significantly reduce risk of later childhood and adult obesity among this population. Interventions that shape parenting behaviors in the areas of sleep and soothing, appropriate and responsive feeding, and routines have been cited as being of particular promise in early obesity prevention [16] and have demonstrated effectiveness in our previous trials [14, 17–19]. However, these interventions have yet to be tested among African American mothers and their infants [16].

Sleep SAAF is a prospective, two-arm, randomized controlled clinical trial evaluating whether a responsive parenting intervention focused on promoting infant sleeping and self-soothing can prevent rapid weight gain during the first 16 weeks postpartum among first-born African American infants. The responsive parenting intervention is being compared to a child safety control intervention. The responsive parenting intervention addresses infant sleep, soothing/crying, and feeding during the first months of life. The safety intervention addresses safe sleep practices as well as other aspects of newborn safety (e.g., food safety, preventing falls, poison prevention, preventing burns, car seat safety). The interventions are delivered at participants’ homes by Community Research Associates (CRAs), who are African American community members from communities similar to the ones in which we are implementing the intervention.

### Conceptual framework

Sleep SAAF’s responsive parenting intervention is grounded in the developmental literature on parenting sensitivities, arguing that parenting that is developmentally appropriate, prompt, and contingent on the infant’s needs [20] can increase infant sleep and self-regulation, and reduce rapid infant weight gain. The intervention is focused on a critical developmental period when plasticity is high. How parents put their infant to bed (e.g., bedtime routines, falling asleep independently) and respond to nighttime awakenings represent important sources of variability in infants’ developing sleep patterns [21, 22]. Because infant sleep bouts are interrupted by waking to be fed, the routines parents establish around sleep can also affect feeding frequency and energy intake, with important consequences for infant weight gain. Similarly, how parents respond to infant crying represents an important source of variability in infants’ developing regulation skills; avoiding the use of feeding as the first response to infant distress by using alternative soothing strategies can increase parental responsiveness to infants’ needs and promote infants’ self-regulation.

**Fig. 1:**
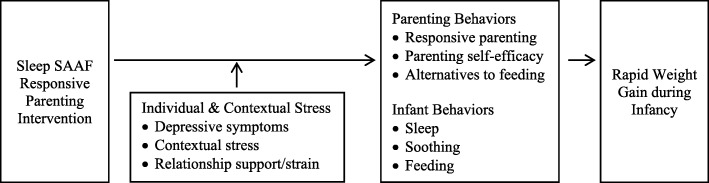
Early factors affecting rapid weight gain among African American infants

Individual and contextual stressors are common among African American mothers and have the potential to affect intervention effectiveness. For example, depressive symptoms are prevalent among African American mothers of infants [23] and are likely to interfere with maternal responsiveness [24]. The broader social context is also likely to pose challenges [25]. The families participating in Sleep SAAF live in communities in Georgia where poverty rates are high and unemployment rates are above the national average [26]. Under conditions of economic distress, parents are vulnerable to anger, low frustration tolerance, and depressive symptoms [27–30], all of which may be consequential for responsive parenting. African American families living in the South must also contend with race-based stressors such as racial discrimination, with negative consequences for depressive symptoms, health, and responsive parenting [31–35]. Lastly, many African American mothers do not have a steady romantic partner at the time of their infant’s birth [36], potentially limiting available social support and increasing parenting stress [37]. We examine each of these stressors as moderators of program effectiveness. Our conceptual framework is shown in Fig. 1.

### Evidence from previous trials

Sleep SAAF builds on our two previous RCTs providing evidence for the efficacy of a responsive parenting program. In a pilot study, Birch and colleagues developed and evaluated the SLIMTIME intervention for primiparous mothers who intended to breastfeed [19]. Participants were predominantly middle income and White. The first component of SLIMTIME, “Soothe/Sleep,” began when infants were 2–3 weeks old and focused on promoting aspects of responsive parenting to promote the development of infant self-soothing and longer sleep duration and to reduce feeding frequency. As predicted, there was evidence of changes in sleeping, feeding, and soothing as a result of the intervention [19, 38]. Infants whose mothers participated in the Soothe/Sleep intervention slept longer at night, had fewer total daily feeds, and had fewer nocturnal feeds from age 3 to 16 weeks compared to control group infants. Infants who received the intervention also gained weight more slowly over the first year, indicating that intervening on sleep and soothing behaviors can reduce the rate of weight gain during infancy.

In a subsequent larger scale randomized controlled trial (INSIGHT), the sample included primiparous mothers intending to formula feed as well as those intending to breastfeed and was predominantly middle income and White [39]. This comprehensive responsive parenting program addressed infant feeding, sleeping and soothing, active social play, emotion regulation, and growth record education, and was taught to mothers at four home visits at 2-, 16-, 28-, and 40 weeks. During the first 16 weeks, program content focused on soothing, sleeping, and breast or bottle feeding; after 16 weeks, the focus was expanded to include introducing solids, table foods, and active social play. Relative to a home safely control, infants in the responsive parenting condition gained weight more slowly than control group infants from birth to 6 months, had lower weight-for-length percentiles at 1 year, were less likely to be overweight at 1 year, and had lower BMI z-scores through age 3 [14, 17]. There were also positive sleep outcomes: infants in the responsive parenting condition had longer nighttime sleep duration, more consistent bedtime routines, and earlier bedtimes, and were more likely to self-soothe to sleep without being fed at 8-, 16- and 40 weeks of age [18].

### Study aims and hypotheses

Sleep SAAF builds on the efficacy of these multi-component responsive parenting programs. Focusing on the initial soothe/sleep component of SLIMTIME and INSIGHT, we test whether a responsive parenting program can promote infants’ sleep and self-soothing and reduce rapid weight gain during the first 16 weeks of life among African American infants born in the South. Our specific aims are:

1. To assess the effects of responsive parenting, focused on infant sleep and soothing, on reducing RWG from 3 weeks to 16 weeks among African American infants. We hypothesized that infants in the responsive parenting condition relative to safety control would show less rapid growth from 3 weeks to 16 weeks.
2. To assess effects of responsive parenting on parental and infant behaviors at age 8 weeks, and whether these mediate effects on infant growth from 3 to 16 weeks. We hypothesized that compared with control mothers, mothers in the responsive parenting condition would report higher levels of responsive parenting, parenting self-efficacy, and greater use of alternatives to feeding to soothe, and lower levels of feeding to soothe at 8 weeks postpartum; infants in the responsive parenting condition would have longer sleep bouts and fewer feedings at age 8 weeks. These outcomes would be linked to healthier patterns of weight gain from 3 weeks to age 16 weeks.
3. To examine moderation of intervention effects by individual and contextual factors. We examined whether individual and contextual factors common among African American mothers moderated the effectiveness of the responsive parenting intervention, increasing our understanding of how these stressors affect the ability of high-risk samples to benefit from preventive intervention.

## Methods/design

### Overall study design

**Fig. 2:**
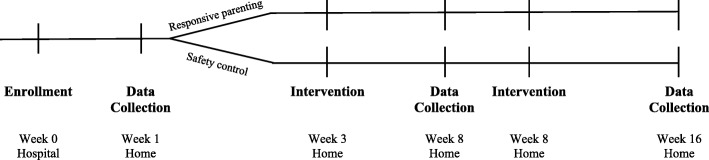
Sleep SAAF study visit schedule

Sleep SAAF is a two-arm randomized, controlled clinical trial evaluating the effectiveness of a responsive parenting intervention relative to a child safety control. Following initial screening and consent in the hospital by the project’s Recruitment Coordinator, mothers and their infants were visited at home by CRAs at 1 week, 3 weeks, 8 weeks, and 16 weeks postpartum for intervention and data collection visits (Fig. 2). To minimize bias during the assessments, separate CRAs completed the intervention visits (at 3 weeks and 8 weeks) and data collection visits (at 1 week, 8 weeks, and 16 weeks). Further, CRAs were trained in (and subsequently delivered) only one of the two study interventions (responsive parenting or child safety).

Funding for this project comes from the National Institute of Diabetes and Digestive and Kidney Diseases (NIDDK; R01DK112874) and the study was registered on www.clinicaltrials.gov (NCT03505203) on April 3, 2018. This study was approved by the Augusta University Institutional Review Board and overseen by a Data Safety Monitor, based on NIDDK guidelines that a Data Safety Monitor is more appropriate for single-site clinical trials that are not masked and are minimal risk, as is the case in the current study. Additional details on the Data and Safety Monitoring Plan are provided in Additional file 1.

### Participants and recruitment

A total of three hundred African American mother-infant dyads will be recruited from the mother/baby nursery at Augusta University Medical Center (AUMC) in Augusta, GA. Recruitment began in the spring of 2018 and will continue through the summer of 2021. AUMC provides comprehensive medical care for the Augusta area and serves as a referral center for the state of Georgia and western South Carolina. All newborns delivered at AUMC were screened for participation. The project’s Recruitment Coordinator had access to electronic medical records systems in order to pre-screen mothers and infants based on the following criteria: (1) full-term infants (> 37 0/7 weeks gestational age), (2) singleton infant; (3) infant > 2500 g at birth; (4) primiparous mother > 17 years of age, (5) mother self-identifying as African American; (6) residence < 75 miles from Augusta; and (7) English speaking. In addition, mother-infant dyads were excluded if there was a congenital anomaly or neonatal physical or metabolic condition that would significantly affect the newborn’s feeding or growth (e.g. cleft palate, complex congenital heart disease); if there were major maternal morbidities or pre-existing conditions that would affect postpartum care or the mother’s ability to care for her newborn (e.g., narcotic drug use, uncontrolled depression causing social service contact); if there was a plan for the newborn to be adopted; or if the family planned to move from the area within four months of delivery.

### Randomization

Participants were randomized to either the responsive parenting intervention or child safety control after completion of the 1-week data collection visit, stratifying on sex-specific birth weight for gestational age (<50th percentile or > 50th percentile) [40] and intended feeding mode (breastfeeding or formula). Randomization was done using a secure Microsoft Excel application prepared by a statistician unaffiliated with the study and administered by the study’s Project Coordinator. Blinding was maintained after allocation only for the CRAs completing the data collection visits. Participants were made aware of their condition, CRAs serving as intervention facilitators were aware of condition because they were only trained in one of the two interventions (responsive parenting or child safety), and study investigators and other project staff (e.g., Project Coordinator, Intervention Coordinator) were aware of condition.

### Responsive parenting intervention

**Fig. 3:**
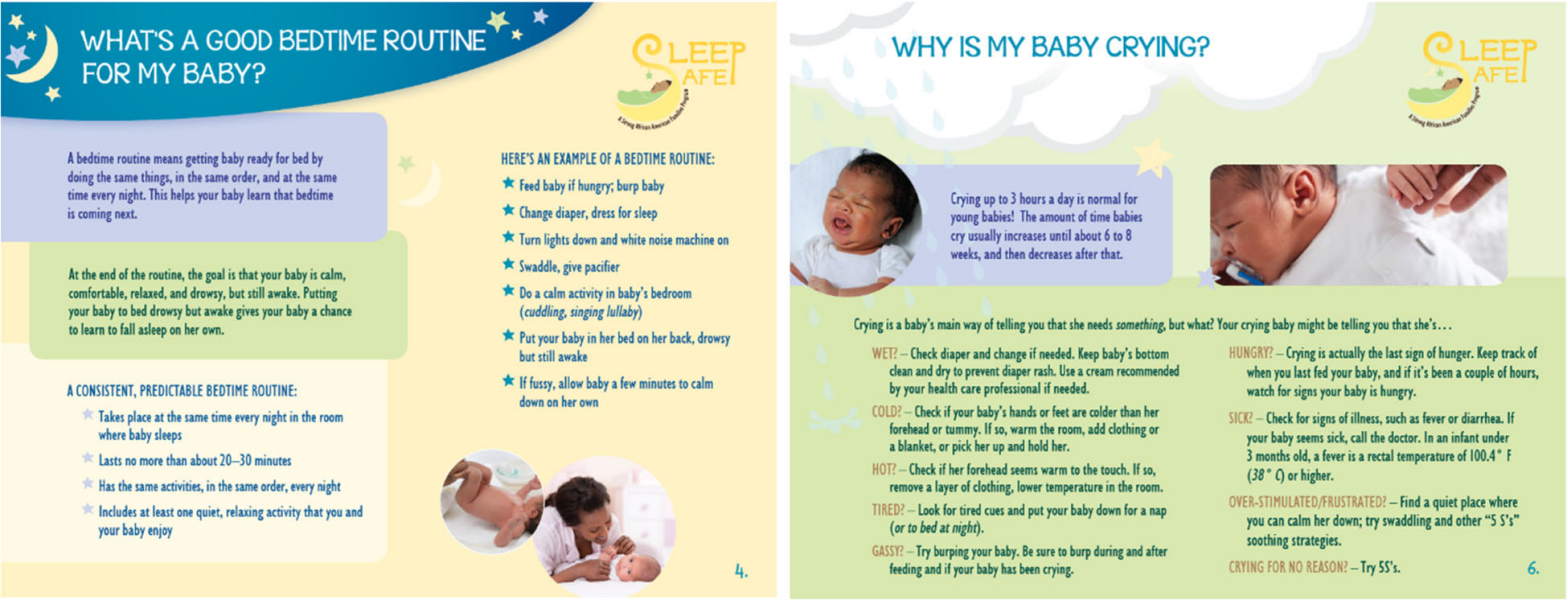
Examples of sleep/soothe responsive parenting messages

The responsive parenting intervention focused on responsive parenting in the context of (1) sleeping, (2) crying, (3) feeding, and (4) playing (Fig. 3). These topics were introduced and discussed in detail at the first intervention visit at 3 weeks postpartum and reviewed at a booster session at 8 weeks postpartum. All materials were tailored for African American families.

#### Sleeping

We provided information about normal sleep patterns in infants, including how many hours of sleep infants typically need and frequency of night waking [41]. We also provided guidance on establishing a consistent bedtime routine that includes putting the infant to bed early, drowsy but awake, and avoiding feeding the infant to sleep or putting the infant to bed with a bottle. We also discussed “dream feeds,” where the mother wakes the baby to feed before the mother goes to bed. We briefly addressed safe sleep strategies to prevent Sudden Infant Death Syndrome (SIDS) using materials from the National Institute of Child Health and Human Development (NICHD) [42]. This guidance included always placing baby on her back, on a firm sleep surface, not smoking around baby, keeping baby’s sleep area close to, but separate from, where mother and others sleep, and avoiding the use of pillows and blankets.

#### Crying

Guidance on crying included information on reasons for infant crying, that crying does not always indicate hunger, how to discriminate hunger from other reasons for infant crying, and how to use alternative soothing strategies other than feeding. Specifically, mothers learned various reasons why babies cry (e.g., wet, cold, hot, tired, gassy, over-stimulated) and that these cries differ in systematic ways, and viewed video clips illustrating these differences. Mothers were also taught soothing strategies that they could use to soothe their crying baby, including Shushing, Swinging, Side/Stomach Position, Sucking, and Swaddling [43]. They also received swaddle blankets and instruction and practiced swaddling their baby. We also highlighted strategies for dealing with night waking to promote self-soothing, including allowing the infant a brief time to self-soothe before intervening to soothe the infant.

#### Feeding

Intervention staff taught mothers to recognize hunger cues (rooting, mouthing, bringing hand to mouth) and fullness cues (letting go of nipple, falling asleep, turning head away, interest in other things). Expectations for typical feeding frequency during the day and night for breastfed and formula fed infants were discussed. Mothers were given education on age-appropriate bottle sizes, milk/formula volumes, use of slow-flow bottle nipples for infants under 4 months to prevent overfeeding or choking, and how to use fullness cues, rather than the amount of milk in the bottle, to determine when to terminate a feeding. Mothers were also advised that breast milk or formula is best for infants of this age, to delay the introduction of solid foods and other beverages until age 6 months, and to avoid adding infant cereal to a bottle.

#### Playing

Lastly, we encouraged mothers to play with their baby every day and to do tummy time with their baby for at least a few minutes each day. We also reviewed strategies to promote mothers’ well-being (e.g., sleeping when baby is sleeping, reaching out to friends and family for support).

### Safety control

Sleep SAAF’s control group received a developmentally-appropriate child safety intervention. The home visits were designed to be equal in length and intensity to the responsive parenting intervention visits and to avoid messages that could impact responsive parenting. At 3 weeks, mothers received an extended version of the safe sleep strategies presentation from NICHD [42], which provided more detail about the Back to Sleep Campaign, SIDS facts and myths, reducing baby’s risk, and the importance of good health care. In addition, information was provided about taking care of a crying baby, finding caretakers for baby, and food safety (e.g., formula and bottle handling, preparation, and storage). At the 8-week intervention visit, control group mothers received guidance on general child safety using materials from Safe Kids Worldwide [44]. This included car seat safety as well as home safety tips such as preventing falls, water safety, poison prevention, fire safety, and preventing burns.

### Measures

To assess intervention impacts on the primary outcome, intermediary behavioral processes, and moderators of intervention effectiveness, data collection included measures divided into the following categories: Anthropometry, Sleep Outcomes, Infant Soothing and Feeding Outcomes, Mothers’ Outcomes, Child Safety Outcomes, Stress-Support Moderators, and Demographics and Health History. As shown in Table 1, many of these measures were assessed multiple times. CRAs also rated coparent involvement in the intervention and implementation quality following the intervention sessions.

**Table 1:** Sleep SAAF measures by time point

Construct	Time points (child age in weeks)			
	1	3	8	16
Anthropometry				
Infant weight, length, and head circumference	X	X	X	X
Mother weight		X	X	X
Father weight (if applicable)		X	X	X
Sleep Outcomes				
Mother report of infant sleep		X	X	X
Mother report of own sleep	X		X	X
Maternal actigraphy			X	
Infant Soothing and Feeding Outcomes				
Infant soothing			X	X
Infant temperament				X
Feeding frequency		X	X	X
Baby’s eating behavior				X
Mothers’ Outcomes				
Parenting self-efficacy			X	X
Maternal feeding practices and beliefs			X	X
Depressive symptoms	X		X	X
Physical health				X
Child Safety Outcomes				
Safe sleep practices	X			X
Child safety practices				X
Stress-Support Moderators				
Maternal personality	X			
Family background	X			
Financial strain/employment	X			
Discrimination	X			
Romantic relationship characteristics	X		X	X
Coparent relationship characteristics				X
Social support	X			
Family routines			X	
Demographics and Health History				
Family demographics, maternal/infant health	X			
Intervention-Related Variables				
Coparent involvement in intervention		X	X	
Implementation quality		X	X	

Self-report data were collected using a secure, web-based survey interface, Qualtrics, on iPads. When literacy concerns arose, CRAs read the questionnaires to mothers, who subsequently entered their answers directly into Qualtrics. Anthropometry measurements were taken and recorded using Qualtrics by the CRAs.

### Outcomes

The primary study outcome was a between-group comparison of infant conditional weight gain (CWG) scores from 3 weeks to 16 weeks, calculated following the method of Griffiths and colleagues [45] and described in our recent work [17]. There were several secondary weight-related outcomes that we compared between groups as well, including differences in change in infants’ weight for age over time and differences between groups in infants’ weight outcomes at age 16 weeks, including BMI z scores, weight-for-age z scores, and weight-for-length z scores.

Additionally, there were several other pre-specified outcomes assessing infant and maternal responses to intervention, including: 1) Maternal report of infant sleep (e.g., sleep duration, night awakenings); 2) Maternal sleep actigraphy (e.g., sleep duration, sleep efficiency, wakefulness); 3) Infant soothing (e.g., mothers use feeding for reasons other than in response to hunger); 4) Infant feeding frequency (e.g., feeding frequency, use of bottle feeding, introduction of solids); 5) Safe sleep practices; 6) Child safety practices; 7) Infant eating behavior; 8) Maternal self-efficacy; 9) Maternal feeding practices and beliefs; 10) Maternal depressive symptoms; and 11) Family routines.

### Sample size and power calculation

The primary outcome for Sleep SAAF is between-group differences in CWG scores from 3 to 16 weeks. We used G Power 3.1.9.2 to estimate the smallest intervention effect size we could detect with 80% power, a 5% two-tailed Type 1 error rate, and two groups of 150 each (assuming 10% attrition over the course of the study) [46]. We achieved 80% power with an effect size *d* of 0.34. Our prior research showed an effect size of approximately 0.4 for infant conditional weight gain at 6 months [17], suggesting that we will have sufficient power in our sample to detect intervention effects.

### Statistical analysis plan

All primary statistical analyses will invoke the intent-to-treat paradigm, analyzing data based on randomized assignment. Missing data will be handled using full-information maximum likelihood methods, which use all available information to estimate parameters, making this approach more efficient and less biased than other methods when data are missing at random [47]. All models considered allow for the inclusion of relevant covariates. Covariates to be considered in refining the analyses, in addition to those described in detail in the following paragraphs, include demographic factors (e.g., maternal employment and hours worked, gestational diabetes), weight-related factors (e.g., maternal pre-pregnancy weight/BMI, gestational weight gain, maternal smoking), and intervention-related factors (e.g., implementation quality, coparent involvement in intervention). We will also examine (1) infant sex as a covariate, given different growth charts for boys and girls [48] and (2) feeding mode as a covariate, given differences in weight gain between breastfed and formula-fed infants [49].

#### Analyses for specific aim 1

As described earlier, our primary outcome is between-group differences in conditional weight gain (CWG) scores from 3 to 16 weeks. We will calculate CWG scores following the method of Griffiths and colleagues [45], as described in our recent work [17]. Scores will be calculated as standardized residuals from the linear regression of weight for age at 16 weeks on weight for age at 3 weeks (the first intervention session), with length for age at birth and 16 weeks and infant age at the 16-week assessment entered as covariates. The CWG score represents the variation in child weight gain not explained by child age, initial length, or initial weight. A CWG score of zero represents the population mean [45]. Positive CWG scores (above the estimated regression) indicate more rapid or faster than average weight gain, while negative scores (below the estimated regression) indicate slower weight gain. We will use a linear mixed-effects model [50] to test whether the responsive parenting group and the safety control group differ in their rapid weight gain.

In addition to conditional weight gain scores, we will examine several other secondary weight outcomes. First, we will examine differences in change in infants’ weight for age over time using growth curve models [51]. Specifically, we will conduct growth curve analyses using hierarchical linear modeling to test whether the responsive parenting and safety control groups differ in their rate of change in weight over time. The model will include intervention group, time of growth measure, and infant weight adjusted for gestational age. These analyses will evaluate whether the responsive parenting group shows less of an increase in weight over time compared to the safety control group. Second, we will examine differences between groups in infants’ weight outcomes at age 16 weeks, including weight-for-age z scores, weight-for-length z scores, and BMI z scores. We include multiple growth outcomes because there is not one universally accepted measure for children younger than age 2 years. To compare 16-week outcomes between the responsive parenting intervention group and the safety control group, we will use linear mixed-effects models that include intervention group and infant weight at age 1 week adjusted for gestational age. These analyses will test whether the responsive parenting group and the safety control group differ in their weight outcomes at age 16 weeks.

#### Analyses for specific aim 2

We will examine parental behaviors (parental self-efficacy, sleep/soothing practices, feeding practices, family chaos) and infant behaviors (sleep, soothing, and feeding) targeted by the intervention that may mediate effects on infant growth. We will examine these effects in several steps. First, we will examine differences between the responsive parenting and safety control groups in these outcomes at two time points post-intervention (8- and 16- weeks postpartum) using the linear mixed-effects model described above. The linear mixed-effects model will include intervention group and infant weight adjusted for gestational age. These analyses will test whether the responsive parenting group and the safety control group differ in these behaviors post-intervention and will be used to identify variables for the formal tests of mediation. Second, we will examine whether parent and infant behaviors mediate infant weight outcomes. We will test these hypotheses using structural equation modeling. The first step in demonstrating mediation is to establish the effects on mediating and distal outcomes, as we described above. We will then specify mediators as indirect effects in a path model. Intervention condition will be dummy coded and specified as a predictor of infant and parental behaviors, which in turn predict infant weight gain and infant weight. The significance of the mediating process will be tested using the Sobel or bootstrapping methods [52].

#### Analyses for specific aim 3

We will examine whether individual (depressive symptoms) and contextual (socioeconomic stressors, race-based stressors, romantic relationship characteristics, coparent relationship characteristics, social support) factors at 1-week postpartum moderate program effects. These factors will be included in the analyses described in the preceding paragraphs to determine whether they moderate intervention effects on weight outcomes and parental and infant behaviors.

## Discussion

Racial disparities in overweight and obesity begin early in development and persist throughout the lifespan. Early intervention efforts are needed to reduce these disparities and related comorbidities. Sleep SAAF builds on the effectiveness of our previous trials [14, 17–19] to test whether a responsive parenting program can reduce rapid weight gain among African American infants. If successful, the program would represent a promising approach to early obesity prevention that could be used in clinical practice and integrated into more comprehensive efforts to promote health and well-being among African American families.

## Additional file

Additional file 1: Data and safety monitoring plan, Research maternal informed consent and parental permission for infant document. (ZIP 477 kb)

## Data Availability

Not applicable.

## Abbreviations

AUMC: Augusta University Medical Center
CRA: Community Research Associate
CWG: Conditional weight gain
NICHD: National Institute of Child Health and Human Development
NIDDK: National Institute of Diabetes and Digestive and Kidney Diseases
RWG: Rapid weight gain
SAAF: Strong African American Families
SIDS: Sudden Infant Death Syndrome
