# Supplementary material for: Sleep SAAF: a responsive parenting intervention to prevent excessive weight gain and obesity among African American infants
Source: BMC Pediatr. 2019 Jul 5;19:224. doi: 10.1186/s12887-019-1583-7 (PMC6610994; doi:10.1186/s12887-019-1583-7)
Supplement: Supplementary file 1 — Data and safety monitoring plan, Research maternal informed consent and parental permission for infant document. (ZIP 477 kb) [file 12887_2019_1583_MOESM1_ESM.zip › Data Safety Monitoring PlanR2.docx]

**Sleep SAAF**

**Data and Safety Monitoring Plan**

**Grant # 1R01DK112874-01**

**October 4, 2018**

1. **Overview**

*Purpose of the Study:*

The present study examines whether providing mothers of newborns with responsive parenting guidance during the first weeks of life to promote infant sleep and soothing can reduce rapid weight gain for African American infants born in low SES contexts, and also examines the risk and protective factors that may affect program efficacy.

*Adherence Statement:*

The Data and Safety Monitoring Plan (DSMP) outlined below for R01DK112874 will adhere to the protocol approved by the Augusta University IRB.

*Overall Framework for Safety Monitoring and Information to be Monitored:*

The data and safety monitoring plan (DSMP) for this intervention trial focuses on close monitoring of infant growth by the principal investigators (PIs) in conjunction with a Data Safety Monitor, along with prompt reporting of adverse events to the NIH/NIDDK and to the Institutional Review Board (IRB) at Augusta University. Because behavioral interventions aimed at reducing rapid weight gain could theoretically result in insufficient weight gain by study participants, infants’ growth will be closely monitored by the investigators, who will be overseen by the project’s Safety Monitor, Dr. Reda Bassali. The decision to include a Data Safety Monitor instead of a Data and Safety Monitoring Board (as in the original proposal), was made in April 2018 in consultation with our Program Officer at NIDDK, Voula Osganian, M.D., based on NIDDK guidelines that a Data Safety Monitor is more appropriate for single-site clinical trials that are not masked and are minimal risk, as is the case in the current study.

1. **Adverse Events**

In this study, an adverse event shall be defined as any detrimental change in the participant's condition, whether it is related to the study interventions, study outcomes, or to another unrelated illness. Adverse effects may be (a) unrelated to the study interventions, (b) potentially related to the study interventions, or (c) related to the study interventions.

*Adverse Events Unrelated to the Study Interventions*:

Adverse events due to illnesses unrelated to study interventions may be grounds for withdrawal if the illness is considered significant by the study investigators or if the participant is no longer able to effectively participate in the study. A significant illness would be one that would compromise the child’s ability to function normally and thrive, such as the diagnosis of a malignancy, illness characterized by growth problems, disease requiring ongoing and intensive treatment, and/or one requiring repeated hospitalizations or physician visits. Subjects experiencing routine, minor, self-limited acute illnesses that typically occur during infancy and do not affect long-term growth will not be recorded, and the child will continue to participate in the study. Examples of minor illnesses include acute otitis media, bronchiolitis, upper respiratory infections, urinary tract infections, and gastroenteritis. Medications for acute, self-limited illnesses such as those stated above will not be recorded, but chronic medication use (> 1 month) will be recorded.

Other adverse events that could affect growth include a milk-protein allergy, other food allergies, or physician-diagnosed gastroesophogeal reflux disease (GERD) requiring medication. Surgical conditions such as intestinal malrotation and pyloric stenosis also would impact infant feeding and weight gain. Therefore, these will be recorded and if the study investigators determine that these conditions can affect long-term growth, the participant will be withdrawn from the study.

Documentation of an adverse event unrelated to study interventions that are not considered minor illnesses of childhood and those that can significantly affect growth will be recorded using Augusta University’s interactive digital form for reporting adverse events

*Adverse Events Related or Potentially Related to the Study Interventions:*

It is theoretically possible that behavioral interventions designed to prevent rapid weight gain during infancy could result in underfeeding by parents and insufficient growth. The NIH-funded studies that serve as the foundation for the current project did not find an association between study interventions and these adverse events. While the results of these previous projects are reassuring, weight status and growth will be closely monitored and evaluated at frequent intervals in the current project in order to monitor the study intervention for potential adverse events. In the current study, there are several ways a potential adverse event related to growth will be identified:

1. Weight-for-age below the 5^th^ percentile using current CDC growth charts, based on WHO data from birth to 2 years.
2. Downward crossing of two major percentile lines on the WHO weight-for-age growth chart statistically evaluated as equal to or greater than a -0.67 Z-score change in order to provide a consistent measure across subjects.

Recognizing that growth monitoring is typically performed at all regularly scheduled primary care appointments, we will also ask mothers if their infant’s PCP expressed concerns that the infant is demonstrating insufficient weight gain. In turn, the study team will determine if the infant meets one of these screening criteria above when assessed at study visits at 1 week, 3 weeks, 8 weeks, or 16 weeks.

During each study visit, home visit staff will assess weight. Within one week of the visit, the Project Coordinator will plot each child’s growth on the CDC growth charts to allow for identification of potentially concerning growth patterns. Dr. Stansfield will be immediately alerted by the Project Coordinator to any concerning patterns (i.e., weight-for-age below the 5^th^ percentile or downward crossing of two major percentile lines). After each child is closely evaluated, Dr. Stansfield will then contact the mothers by phone when necessary (i.e., when there is a potential adverse event related to infant growth), to discuss their infants’ growth and whether a visit with the infant’s primary care provider is necessary. If Dr. Stansfield is unable to reach the infant’s mother and/or if the mother requests it, Dr. Stansfield will contact the infant’s primary care provider directly. Dr. Stansfield will document these calls as they occur as a research note. Dr. Stansfield will review growth charts of all active study participants at least twice monthly.

For any individual child that meets initial screening criteria for growth concerns, numerous factors will be considered in determining whether the child’s growth is problematic and/or related to study interventions. Examples of such factors include genetic potential based upon parental size, the participant’s linear growth, feeding mode (breast milk vs. formula), and interval illnesses.

The study’s informed consent document will include information indicating that the study team will communicate with the participant’s PCP if necessary; all infants are required to have a PCP prior to discharge from the hospital and are expected to receive normal medical care (e.g., well-baby visits) throughout the study as is typical for infants. Doing so will allow for open lines of communication between the study team and the PCPs in the event of concerns related to growth. If either the primary care provider or the study investigators believe that it is possible that these growth patterns are a negative result from study participation, the child will be withdrawn from the study.

*Expected Rates for Failing Screening Criteria for Adverse Events Potentially Related to Study Interventions:*

Using standard population distributions, it is expected that 5% of children will be below the 5th percentile on the weight-for-age growth chart. For downward crossing of two major centile lines, it is expected that this will occur with some frequency given the well-established phenomenon of regression to the mean, which suggests that infants born at the higher percentiles have a higher probability of moving downward to the population mean. The results of our pilot study suggest that 15% of participants may experience this, and that will serve as the cut-off alarm value for this study. Combined, these two screening criteria allow for up to a 20% adverse event rate for those events related or potentially related to the study interventions.

*Process by which Adverse Events Will be Managed and Reported:*

As outlined above, we will continuously monitor adverse event rates in all participants. Dr. Stansfield will have primary responsibility for reporting Adverse Events, Serious Adverse Events, and Unanticipated Problems to the Augusta University IRB, the study’s Safety Monitor, and the NIH/NIDDK as required. If Dr. Stansfield is unable to fulfill these duties, PIs Birch and Lavner will have secondary responsibility for reporting.

1. **Safety Review Plan and Monitoring**

*Frequency of Monitoring:*

As described above, the Project Coordinator will plot each child’s growth on the CDC charts within one week following study visits to allow for identification of potentially concerning growth patterns. Dr. Stansfield will review the growth charts of active study participants at least twice monthly to allow for close monitoring and contact mothers as necessary. Safety reports will be sent to Dr. Stansfield, the PIs, and the Safety Monitor (Dr. Reda Bassali) twice per year. The Project Coordinator will be responsible for assembling the data and producing these reports in conjunction with the study statistical team, as well as assuring that all parties obtain copies of these reports.

*Plans for Interim Analysis*:

We plan to collect adverse event data from the intervention and control groups as they occur, and will report them twice per year as necessary. The investigators will not be blinded to the treatment groups. Therefore, it seems that blinding the treatment status on the biannual reports will be unnecessary. We plan to present un-blinded adverse events data to Dr. Stansfield, the PIs, and the Safety Monitor throughout this trial. We do not expect adverse event rates to be different between treatment groups or exceed that found in usual clinical care.

*Stopping Rules*:

As outlined above, we will continuously monitor adverse event rates in all participants. The study investigators, together with the Safety Monitor, will alert the IRB and the NIH/NIDDK if a greater than or equal to 20% adverse event rate potentially due to study interventions should occur in the treatment group.

Any participant with a serious adverse event related to the study interventions will be discontinued from the study. Should insufficient weight gain be detected and the cause of this weight gain is related or potentially related to the study interventions as determined by Dr. Stansfield and the PIs, the participant will be discontinued from the study. Other non-minor concurrent illnesses or major changes in the family social environment would also lead to discontinuation as determined by the investigators.

As described in detail above, we will closely monitor rates of insufficient growth for individual infants and by study treatment group. In addition to individual level monitoring, the study statistician will analyze rates of insufficient growth by treatment group on a biannual basis if necessary. If there are significantly more children with inadequate growth in the intervention group at any point, these data will be presented to the Augusta University and UGA IRBs, the study’s Safety Monitor, and the NIH/NIDDK so that a decision can be jointly made as to whether the trial needs to be suspended.

We acknowledge that there are other situations that could occur that might warrant stopping the trial, and these concerns will be discussed by Dr. Stansfield, the study PIs and the Safety Monitor as needed.

1. **Data Quality and Management**

The biannual reports will also include information on Data Quality and Management. This information will include statistics on enrollment, including number of participants screened for eligibility, number of participants excluded and reasons for exclusion, number eligible, number declining to participate and reasons for declining, number of participants enrolled, number of participants withdrawn before randomization, number of participants randomized into intervention and control groups, number of participants withdrawn after randomization and reasons for withdrawing, and the number of participants lost to follow-up. We will also include statistics on retention, including home visit participation rates at 3 weeks, 8 weeks, and 16 weeks. Lastly, we will include statistics on data completeness/missing data, including how many participants have weight data at 1 week, 3 weeks, 8 weeks, and 16 weeks, as well as how many participants have self-report data at these time points. For enrollment, retention, and data completeness/missing data, we will include information from that biannual period as well as cumulative data from the study. As with the safety data, the Project Coordinator will be responsible for assembling these data and producing these reports in conjunction with the study statistical team.

1. **Individuals Responsible for Monitoring and Advising the Appointing Entity**

The Safety Monitor for the study will be Reda Bassali, M.D., Associate Professor of Pediatrics at Augusta University. Dr. Bassali is Division Chief of the Department of General Pediatrics at Augusta University and has the necessary expertise and experience to serve as an objective Safety Monitor for this trial. Dr. Bassali is not involved in or affiliated with the grant in any capacity other than in his role as Safety Monitor. He has agreed to serve as the Safety Monitor for this project.

**Checklist for Safety Monitor to Complete After Reviewing the Quarterly Data and Safety Report**

1. Were there any adverse events related to the study treatments? Yes ____ No _____
2. Were participants failing screening criteria for growth concerns evaluated

and reviewed with the infant’s mother? Yes ____ No _____

1. Were more than 5% of the study participants below the 5th percentile on

the weight-for-age growth chart? Yes ____ No _____

1. Is there a significant difference in proportion of children below the 5th percentile on the weight-for-age growth chart between treatment groups? Yes ____ No _____
2. Are there more than 15% of participants with downward crossing of two

major centile lines between two study visits on the weight-for age growth

chart? Yes ____ No _____

1. Is there a significant difference in proportion of children with downward

crossing of two major centile lines between two study visits on the weight-

for-age growth chart between treatment groups? Yes ____ No _____

1. Is the overall adverse event rate potentially due to study interventions

above 20% in either group? Yes ____ No _____

1. Are there any other situations that have occurred in the trial since the last

safety report that warrant triggering a study investigator conference call or

contact with the Augusta University IRB or NIH/NIDDK? Yes ____ No _____

If yes, please explain:

1. Are there any concerns about study progress based on participant

enrollment and/or retention? Yes ____ No _____

1. Are there any concerns about data quality or missingness? Yes ____ No _____

1. Based on any of the replies above, is there any need to communicate

with the Augusta University IRB and/or the NIH/NIDDK regarding data

monitoring or safety of participants? Yes ____ No _____

1. Additional comments:

____________________________________________________________________________________

Signature of Safety Monitor Date
