# Supplementary material for: Sleep SAAF: a responsive parenting intervention to prevent excessive weight gain and obesity among African American infants
Source: BMC Pediatr. 2019 Jul 5;19:224. doi: 10.1186/s12887-019-1583-7 (PMC6610994; doi:10.1186/s12887-019-1583-7)
Supplement: Supplementary file 1 — Data and safety monitoring plan, Research maternal informed consent and parental permission for infant document. (ZIP 477 kb) [file 12887_2019_1583_MOESM1_ESM.zip › Informed ConsentR2.pdf]

Approval Date:	Expiration Date:	IRBNet ID:	
February 27, 2019	October 30, 2019	981204-12	AUGUSTA UNIVERSITY IRB APPROVED

Participant's Name: ___________________________

Participant's Medical Record Number: ___________

Augusta University

Research Maternal Informed Consent and Parental Permission for Infant Document

Sleep-Safe: A Strong African American Families Study

**Principal Investigator:** Brian Stansfield, MD, Assistant Professor, MCG	**Principal Investigator telephone number (available 24/7 and for emergencies):** 706-721-2331, bstansfield@augusta.edu
**Sub Investigator(s):**	**Faculty Advisor:**
**Sponsor:** National Institutes of Health, National Institute of Diabetes and Digestive and Kidney Diseases	**Other Study Contact Numbers:** Jessica Smith 706-542-7611

You and your baby are being asked to take part together in this research study about healthy, safe sleep patterns among African American infants and mothers as normal/healthy volunteers.

The purpose of this document is to:

- Explain your rights and responsibilities
- Explain the purpose of the study
- Describe what will happen if you decide to take part in this study
- Explain the potential risks and benefits of taking part in the study

Participation in research studies is voluntary. Please read this consent form carefully and take your time making your decision. As the study staff discusses this consent form with you, please ask them to explain any words or information that you do not clearly understand. You are encouraged to talk with your family and friends before you decide to take part in this study.

Please tell the study staff if you are taking part in another study.

Why is this study being done?

Getting enough sleep is important to good health and quality of life, and getting enough sleep is especially hard when you have a new baby. The purpose of this study is to learn more about effective ways to increase healthy, safe sleep patterns among African American infants and mothers. African American babies are at a greater risk for health problems resulting from unsafe sleep practices. In addition, African American parents and babies are less likely to get enough sleep. Not getting enough sleep can contribute to multiple health problems for both babies and parents, both in the short term and in the future. This project is designed to find out if teaching mothers about ways to soothe their crying babies can help reduce infant crying and promote safe sleep habits among African American infants.

How long will I be in this study?

Your active participation with your baby in this study is expected to take 16 weeks, or until your baby is about 4 months old. You can choose not to be in the study or stop participating at any time without penalty or loss of any rights or benefits you are entitled to. Please talk to the study staff first before you stop participating in the study.

What will happen to me in the study?

<u>Summary of Visits:</u>

If you decide to participate, you will be visited at your home by a member of our study team a total of four times over the course of the study, when your infant is 1, 3, 8, and 16 weeks old.

<u>Screening/Enrollment at AU Hospital Newborn Nursery:</u>

<u>After agreeing to participate by signing the consent form,</u> we will complete the first part of the study procedures while you and your baby are here at the hospital:

1. Study explanation
2. Sign informed consent and parental permission for newborn
3. Recruitment coordinator will record information from AU medical chart (race, maternal age, gestational age, weight at delivery and (self-reported) pre-pregnancy weight)
4. Complete a form sharing contact information
5. Coordinator will measure weight (kg) and height (cm) of mothers and fathers (if father not present, will attempt to measure at subsequent visit)
6. Coordinator will measure infant length (cm), weight (g), head circumference (cm), mid-thigh circumference (cm).

7. Schedule the first research visit at the home for 1 week postpartum

Home Visits:

Each home visit will take between 1-2 hours. We will schedule your first visit for when your baby is ~1 week old. During this visit, you will complete a series of questionnaires and you and your baby will be measured.

1 Week Initial Visit:

1. Provide basic infant sleep safety information and materials
2. Complete study related surveys
3. Measure infant weight and length
4. Measure maternal weight

After that visit, the study team will use a computer program to randomly assign (similar to flipping a coin) you and your baby to one of the 2 study groups.

The 2 groups are:

1) Sleep Soothe where parents are given information on how to respond to

their baby's cues related to sleeping and fussiness.

2) Sleep Safe where parents are given information on a safe sleep environment, as well as other strategies you can use to keep your baby safe.

You will not know which group you and your baby are in when you sign this form, agreeing to be in the study. You will find out which group you are in at your second home visit. Throughout the visits you will learn ways to care for your baby, especially about sleep. Information about sleep will be different depending on which study group you and your baby are assigned to. The study associate will also ask to walk through your home with you to look at where your baby sleeps and other areas where you and your baby spend time. You do not have to allow the study associate anywhere in your home that you are not comfortable with her entering.

3 Week Study Visit:

1. Deliver study information and materials
2. Complete study surveys
3. Measure infant weight and length
4. Measure maternal weight

8 Week Study Visit:

1. Deliver study information

2. Complete study surveys
3. Provide activity monitors/diaries for mother and baby to be worn for one week, then picked up by study staff
4. Measure infant weight and length
5. Measure maternal weight

<u>12 Week Study Phone Call:</u>

1. Scheduled phone call to check in and remind of 16 week assessment.

<u>16 Week Final Visit:</u>

1. Complete study surveys
2. Measure infant weight, length, head circumference, mid-thigh circumference
3. Measure maternal weight

At each visit, we will measure your weight and your baby's weight and length. Your baby's growth measurements will be shared with you and also monitored by the study team. If any of your baby's measurements suggest a cause for concern related to his or her growth, the study's Principal Investigator (listed above) will notify you and may also notify your baby's Primary Care Provider (PCP). As part of your enrollment information for this study, we will ask you to provide the name and contact information for your baby's Primary Care Provider (PCP).

What tests and/or extra tests will I have if I take part in this study?

**Normal clinical care procedures**	**Research procedures done only because of study**
None.	All procedures are solely for research purposes.

What are the risks of being in this study?

As a result of your participation in this study, you and/or your baby may have the following side effects and/or discomforts from:

Data Collection:

Body measurements: There is a low possibility that you or your baby may experience minor physical discomfort or stress from the height (one time for mother), infant length and weight

measurements we will conduct. To minimize any risk of stress, measurements will be taken in a private room in the hospital and in the privacy of your home at each visit.

Questionnaire completion: There is a low possibility that you may experience minor psychological discomfort or stress from the questions about your romantic relationships, co-parenting, depressive symptoms, diet, infant/child feeding practices, behavior and health history. This information however, is important so that we may correctly evaluate how these influence your health during the first few months postpartum. To minimize the discomfort or stress, data collection will take place in private rooms or in the privacy of your own home.

You should discuss these with the study doctor and your regular health care provider if you choose to do so.

There may be more risks that are not known or not expected.

The study staff will tell you about new information that may affect your health, welfare, or willingness to stay in this study.

Permission to be photographed:

Please provide initials below if you consent for photography and subsequent use of your image and that of your baby for research-related purposes, such as presentations and publications related to this research study. You may still participate in this study even if you are not willing to have your photograph taken.

_____ I am willing to have my/my baby's photograph taken and used as described above
_____I do not want to have my/my baby's photograph taken and or used as described above

Will I benefit from this study?

The possible benefits of this study are:
Participants may experience improved health outcomes such as improved sleep and soothing during the first 16 weeks of life. All participants may benefit from sleep safety and information about how to promote child safety at home. There may be other positive effects on babies' and mothers' functioning.

The likelihood of you benefitting from this study is low.

Who will see my study information?

Study team members, the sponsor of the study, and their representatives will be able to see your study information. Your records may also be reviewed in order to meet federal or state regulations. Reviewers may include the Augusta University Institutional Review Board (the committee who oversees safety of volunteers in research studies), institutional officials, and outside agencies, such as the Food and Drug Administration (FDA).

How will you keep my study information confidential?

Any study information about you and your baby will be kept private and will only be given out with your permission. Once complete, the results of this study will be available online at https://clinicaltrials.gov/. If the results of this study are published, your names will not be used. Your research records will be private to the extent allowed by law. In order to make sure the research is done properly, the Institutional Review Board (IRB – the Board that oversees research at Augusta University) may need access to information about your participation in this study. If you sign this consent form, you are giving us permission to collect, use and share your health information and your baby's health information that is collected for the study.

Upon enrollment, you and your baby will be assigned a study ID number which will serve as a code associated with all collected data. This code will be linked to you and your baby in only one document, the project directory, which will be password-protected using an approved data management software system. Only pertinent study team members will have access to any and all data using a secure internet connection. Electronic and paper versions of de-identified data will be stored separately from any files that may contain subject names. You and your baby will not be identified in study records or publications disclosed outside Augusta University.

This research is covered by a Certificate of Confidentiality from the National Institutes of Health. The researchers with this Certificate may not disclose or use information, documents, or biospecimens that may identify you in any federal, state, or local civil, criminal, administrative, legislative, or other action, suit, or proceeding, or be used as evidence, for example, if there is a court subpoena, unless you have consented for this use. Information, documents, or biospecimens protected by this Certificate cannot be disclosed to anyone else who is not connected with the research except

- if there is a federal, state, or local law that requires disclosure (such as to report child abuse or communicable diseases but not for federal, state, or local civil, criminal, administrative, legislative, or other proceedings, see below);
- if you have consented to the disclosure, including for your medical treatment; or,

- if it is used for other scientific research, as allowed by federal regulations protecting research subjects.

Sharing of information must be done for audit or program review if needed by the group that is paying for this study or for information that is needed by the Food and Drug Administration (FDA), or for other auditing groups.

You should understand that a Certificate of Confidentiality does not prevent you from voluntarily releasing information about yourself or your involvement in this research. If you want your research information released to an insurer, medical care provider, or any other person not connected with the research, you must provide consent to allow the researchers to release it. This means that you and your family must also protect your own privacy.

The investigator can still do what is needed, including reporting to local legal groups (police, etc.), to prevent serious harm to yourself or others.

Because this study is focused on families with growing children, it is possible the researcher will conduct follow-up studies after this one is completed. If you would like to be involved in future studies as a follow-up to this one, the researcher may keep your records in order to contact you in the future. Please select and initial if you would like the researcher to keep your information and contact you about future studies. Your participation in future studies will be voluntary and you can change your mind about taking part at any time.

_____ Yes, please keep my information in the project directory and contact me about follow-up studies to this one.

_____ No, please destroy my information in the project directory at the end of this study and do not contact me for follow-up studies to this one.

What are my costs (what will it cost me) for taking part in the study?

It will not cost you anything to take part in the study other than basic expenses like transportation.

Will I be paid for participation in this study?

You will receive payments for taking part in this study to compensate you for your time and effort. Payment will be in the form checks from the University of Georgia and will be delivered at the end of each study visit listed below.

Compensation Schedule
1 Week Visit: $50
3 Week Visit: $50
8 Week Visit: $75, $25 at pickup of activity monitor
16 Week Visit: $100

If you do not finish the study, we will compensate you for the visits you have completed. You will get $300 total, if you complete all study visits.

In order to process your payments, the researchers need to collect your name and mailing address on a separate form. This form will be sent to the UGA Center for Family Research business office. The researchers have been informed that this office will protect your private information and will keep this confidential by storing in a secured location.

What happens if I am injured or hurt because I took part in this study?

The researchers will exercise all reasonable care to protect you and your baby from harm as a result of your participation. If you think that you or your baby have suffered a research related injury seek medical care right away and contact Drs. Brian Stansfield 706-721-2331 or Leann Birch right away at or 706-542-7611. In the event that this research related activity results in an injury, treatment will be made available including first aid, emergency treatment, and follow-up care as needed. Cost for such care will be billed in the ordinary manner to you or your insurance company. No reimbursement, compensation, or free medical care is offered by Augusta University (AU), AU Medical Center, AU Medical Associates, AU Dental Associates, AU Nursing Associates, Inc., AU Health Professions Associates, Inc. collectively designated AU Affiliates, The University of Georgia, or any other facility involved with this study. You do not give up your legal rights by participating in this study.

Who can answer my questions about this study?
You can ask questions about this study at any time. Please contact the study staff listed on page 1 of this document if you have questions about:

- Study procedures or treatments
- Reporting an illness, injury or other problem
- Leaving the study before it is finished
- Expressing a concern about the study
- Any other questions you may have about the study

Who can I contact to discuss my rights, problems, concerns, questions, or complaints I have as a study participant?

Contact the Augusta University Institutional Review Board at (706) 721-1483.

Could there be any harm to me if I decide to stop participating in the study before it's finished?

If you decide to stop taking part in the study, the study staff will discuss ways to safely remove you and your baby from the study. You should follow the instructions the study staff gives you.

Can I be removed from the study?

Yes, you and your baby may be removed from the study if:

- The sponsor or study doctor decides to stop the study.
- The study doctor stops you or your baby from taking part in the study for your safety.
- You or your baby are not eligible to take part in the study.
- Your condition changes and you need treatment that is not allowed while you are taking part in the study.
- Your baby's condition changes and you need treatment that is not allowed while you are taking part in the study.
- You do not follow the instructions from the study staff.

Authorization to Use or Disclose (Release) Health Information that Identifies You for a Research Study

If you sign this document, you give permission to Augusta University and AU Affiliates to use or release your health information and your baby's health information that identifies you for the study described earlier in this document.

The health information Augusta University and AU Affiliates may use or release for this study includes information in your medical record, information in your baby's medical record, results of physical exams, medical history, lab tests or certain health information indicating or relating to your conditions.

The health information listed above may be used by and/or released to the following, as applicable:

- Researchers and their staff;
- The sponsor of the study including its agents such as data storage banks or contract research organizations monitoring the study;

- Other institutions and investigators participating in the study;
- Data Safety Monitoring Boards;
- Accrediting agencies;
- Clinical staff not involved in the study whom may become involved if it is relevant;
- Health insurers or payers in order to secure payment for covered treatment;
- Parents/Guardians of children younger than 18 years
- Federal/state agencies and Augusta University and AU Affiliates committees having authority over the study. These may include, but are not limited to:
    - The Institutional Review Board (IRB) overseeing this study;
    - Committees with quality improvement responsibilities;
    - Office of Human Research Protections;
    - Food and Drug Administration;
    - National Institutes of Health;
    - Other governmental offices as required by law.

Augusta University and AU Affiliates are required by law to protect your health information. By signing this document, you authorize Augusta University and AU Affiliates to use and/or release your health information for this research.

Once your information has been released outside Augusta University and AU Affiliates, it may no longer be protected by federal laws and regulations and might be disclosed by the persons or institutions receiving the information.

Augusta University and AU Affiliates may not withhold treatment whether or not you sign this Authorization.

You may change your mind and take back (revoke) this Authorization at any time. If you revoke this Authorization, Augusta University and AU Affiliates may still use or release health information and any data and/or specimens already obtained about you as necessary for this study. If you revoke this Authorization, you cannot continue to participate in this study. To revoke this Authorization, you must write to the Principal Investigator listed at the top of this document.

You may not be allowed to see or copy the study information described on this Authorization as long as the study is in progress. Feel free to ask the study staff if this applies to this study. When the study is complete, you have a right to request a copy of your personal health information collected for the study.

Your health information will be used or disclosed when required by law. Your health information may be shared with a public health authority that is authorized by law to collect or receive such information for the purpose of preventing or controlling disease, injury or disability and for conducting public health surveillance, investigations or interventions. No publication or public presentation about the study will reveal your identity without another signed authorization from you.

You will be given a copy of this Authorization. This Authorization does not have an expiration date. If you have questions or concerns about this Authorization or your privacy rights, please contact the Augusta University and AU Affiliates Privacy Officer at (706) 721-5631 or the Toll Free Hotline, 1-800-576-6623.

Regulations require that you be given a copy of the Augusta University and AU Affiliates Notice of Privacy Practices describing the practices of Augusta University and AU Affiliates regarding your health.

STATEMENT OF CONSENT

I have read this form and the information in it was explained to me. My taking part in the study is voluntary. All of my questions were answered. I will receive a copy of this form for my records. I agree to take part in this study. **I am not giving up my legal rights by signing this form.**

____
Participant's Name (print)

___________________________________ ____________________
Participant's Signature Date /Time (00:00)

INVESTIGATOR STATEMENT

I acknowledge that I have discussed the above study with this participant and answered all of his/her questions. They have voluntarily agreed to participate. I have documented this action in the participant's medical record source documents or research chart source documents, as applicable. A copy of this signed document will be placed in the participant's medical record or research chart, as applicable. A copy of this document will be given to the participant or the participant's legally authorized representative.

____

Printed name of Investigator obtaining consent

____ ____________________

Signature of Investigator obtaining consent Date /Time (00:00)
